# In‐silico analysis of the strigolactone ligand‐receptor system

**DOI:** 10.1002/pld3.263

**Published:** 2020-09-15

**Authors:** Marco Bürger, Joanne Chory

**Affiliations:** ^1^ Plant Biology Laboratory Salk Institute for Biological Studies La Jolla CA USA; ^2^ Howard Hughes Medical Institute Salk Institute for Biological Studies La Jolla CA USA

## Abstract

Strigolactones (SLs) are a diverse class of butenolide‐bearing plant hormones associated with several processes of major agricultural concern. SLs initiate symbiosis between plants and arbuscular mycorrhizal fungi, cause germination of crop‐devastating parasitic plants, and inhibit shoot branching in vascular plants. SLs are perceived by dual receptor‐hydrolase proteins, and capturing the intact ligand inside the receptor remains a key challenge for structural biologists. In addition, many discovered SLs are hard to obtain and too unstable to work with. In a computer‐based approach, we investigated the interaction of 20 different SL molecules with nine crystal structures of SL receptors. Our results suggest an important role of the active site for ligand binding and orientation, and that the parasitic plant *Striga hermonthica* has developed both promiscuous and type‐specific SL receptors as part of its host recognition strategy.

## 1 INTRODUCTION

Strigolactones (SLs) are a class of terpenoid‐derived compounds that were first identified as (+)‐strigol, a stimulant for the germination of seeds from the parasitic plant *Striga* (Cook, Whichard, Turner, Wall, & Egley, 1966). It was later discovered that they stimulate hyphal branching in arbuscular mycorrhizal fungi (Akiyama, Matsuzaki, & Hayashi, 2005), a key step in the initiation of symbiosis with their host plants. Later, with the discovery that SLs regulate shoot branching in vascular plants, it became clear that these molecules have a role as endogenous hormones inside the plant body (Gomez‐Roldan et al., 2008; Umehara et al., 2008).

SLs typically include a tricyclic ABC part that is connected to a butenolide D‐ring via an enol ether bridge. This connection is conserved in the 2′*R* configuration and is required for biological activity (Flematti, Scaffidi, Waters, & Smith, 2016; Scaffidi et al., 2014). Two different configurations between the B and C ring ultimately gave rise to two different canonical SL families: strigol‐ and orobanchol‐types. In addition, some SLs lack the A, B or C ring but retain the enol‐ether‐D‐ring, and they are called non‐canonical SLs, which include several SL precursors (Figure S1). Plants often exude a cocktail of SLs into the soil and both canonical and non‐canonical SLs stimulate the germination of parasitic plants (Yoneyama et al., 2018). Some plants such as rice and tomato only produce orobanchol‐type SLs (Xie et al., 2013; Yoneyama et al., 2018).

SLs are perceived by dual receptor‐hydrolase proteins with low substrate turnover (de Saint Germain et al., 2016; Hamiaux et al., 2012; Zhao et al., 2013) that belong to the α/β hydrolase superfamily and convergent evolution of these receptors has driven host detection in parasitic plants (Conn et al., 2015). The SL receptor DWARF14 (D14) was first identified as a component of the SL signaling pathway from an SL‐insensitive mutant of rice, *d14* (Arite et al., 2009). Studies of the *Petunia hybrida* D14 homolog DAD2 then provided direct evidence that the protein is an SL receptor (Hamiaux et al., 2012). After SL binding, the receptor forms a complex with the F‐box protein DWARF3 (D3) and the transcriptional repressor DWARF53 (D53), leading to degradation of D53 by the proteasome. SLs thereby abrogate the repressing activity on the SL signaling pathway (Zhou et al., 2013). Due to the catalytic activity of the SL receptor, it has been challenging for structural biologists to obtain a receptor structure with an intact ligand and the binding mechanism itself is part of an ongoing discussion (Bürger & Chory, 2020; Marzec & Brewer, 2019). A covalently bound hydrolysis intermediate at the histidine residue of the active site (histidine‐butenolide complex) was reported by several groups (Bürger et al., 2019; de Saint Germain et al., 2016; Yao et al., 2016, 2017). One of these studies (Yao et al., 2016) reported a crystal structure that contained a hydrolysis intermediate simultaneously linked to the serine and histidine residues of the active site of AtD14, which the authors named “covalently linked intermediate molecule” (CLIM). However, alternative interpretation for the X‐ray data has been suggested, such as the ligand being an iodide ion (Carlsson, Hasse, Cardinale, Prandi, & Andersson, 2018) or a histidine‐butenolide complex (Bürger & Chory, 2020). Later, is was shown that D14 can be catalytically arrested by the F‐box protein D3 via D3′s C‐terminal helix to prevent premature SL hydrolysis. Binding of the transcriptional repressor D53 to D3 causes D3 to retrieve its dislodged helix, restoring the catalytic activity of D14 (Shabek et al., 2018). In addition, a recent study has concluded that the intact SL molecule possibly triggers the active D14 signaling state (Seto et al., 2019).

In addition to the incomplete understanding of the binding mechanism, access to many SL molecules is limited because they are obtained from their source organism or are chemically synthesized with limited yields that make receptor binding studies difficult. Furthermore, many SLs are challenging to work with due to their chemical instability (Yoneyama, 2020). A detailed understanding of ligand specificities in different SL receptors would be of great help for a better understanding of host specificity of parasitic plants and for the development of specific receptor inhibitors that could serve as agrochemicals.

Here, we report the results of an in‐silico study in which we investigated the interaction of 20 different SL molecules with nine different structures of SL receptors through molecular docking and molecular dynamics (MD). We suggest that a flexible ligand‐binding pocket allows the orientation of SL molecules, driven by the active site, and that the parasitic plant *Striga hermonthica* has developed both promiscuous SL receptors and those that recognize specific types of SLs.

## 2 MATERIALS AND METHODS

### 2.1 Analyses of protein surface hydrophobicity

For analysis and visualization of protein surface hydrophobicity, we used CCP4mg (McNicholas, Potterton, Wilson, & Noble, 2011) and its build‐in GRID (Goodford, 1985) algorithm. GRID parameterizes hydrophobicity by evaluating the interaction of a water probe molecule with all surrounding atoms, rather than roughly classifying amino acids as either hydrophobic, aliphatic or charged/hydrophilic. For example, tryptophan is often considered to be a hydrophobic amino acid, but it has the capacity to form polar interactions through its N^ε^ atom in the indole plane, therefore, depending on the orientation of its side chain toward a ligand, tryptophan may be considered hydrophilic. (Gruber, Zawaira, Saunders, Barrett, & Noble, 2007).

### 2.2 Molecular docking

SL molecules were drawn using ChemDraw (PerkinElmer), converted into PDB files with Chem3D (PerkinElmer), and prepared as PDBQT files using AutoDockTools (Morris et al., 2009). SL receptors were downloaded from the Protein Data Bank and converted into PDBQT files with AutoDockTools. Docking was performed with AutoDock Vina with the following box sizes used (*x*, *y*, *z*): 20, 20, 30 for AtD14; 30, 20, 20 for OsD14; 20, 26, 20 for PhDAD2; 30, 20, 20 for ShD14; 20, 20, 30 for ShHTL1; 30, 20, 20 for ShHTL4; 20, 20, 30 for ShHTL5; 20, 20, 30 for ShHTL7; 20, 20, 30 for ShHTL8. Centers were (*x*, *y*, *z*): −4.556, 4.65, 55.232 for AtD14; 27.259, 14.911, 18.881 for OsD14; −39.109, −37.336, 11.842 for PhDAD2; 24.297, 22.909, 71.445 for ShD14; 19.419, 4.403, 17.932 for ShHTL1; −9.18, −24.374, 1.424 for ShHTL4; −12.748, 26.29, −8.368 for ShHTL5; −20.458, −16.024, −20.27 for ShHTL7; 18.786, −7.138, 4.705 for ShHTL8. Results were analyzed with Phyton Molecule Viewer and visualized using CCP4mg. Distances between atoms were measured with Coot (Emsley, Lohkamp, Scott, & Cowtan, 2010). Affinities are listed in Table S1.

### 2.3 Molecular dynamics

Molecular dynamics (MD) simulations were carried out with GROMACS 2020.1 (Abraham et al., 2015). The receptor proteins were processed with the CHARMM36 all‐atom force field (Best et al., 2012) and ligand atomic coordinates from the docking results were processed through CGenFF (Vanommeslaeghe et al., 2010). The total number of atoms in the simulated system was immersed in a dodecahedron‐shaped box. The solvated system was neutralized by adding Na^+^/Cl^−^ ions in the simulation After energy minimization, successive steps of NVT and NPT (300 K, 1 bar) MD were performed. MD production simulations were performed for 10 ns.

### 2.4 Protein structures

The following crystal structures were used from the Protein Data Bank (PDB): 4IH4 (AtD14), 3W04 (OsD14), 4DNP (PhDAD2), 5Z7Z (ShD14), 5Z7W (ShHTL1), 5Z7X (ShHTL4), 5CBK (ShHTL5), 5Z7Y (ShHTL7), 6J2R (ShHTL8).

## 3 RESULTS

### 3.1 Validation of docking results with existing receptor‐ligand complex structures

To evaluate the reliability of our molecular docking procedure, we carried out docking experiments with existing ligand‐receptor structures and compared our results to the experimental crystal structures. We used the crystal structures of PhDAD2 in complex with glycerol (PDB code 4DNP (Hamiaux et al., 2012)), and a quinazolinone derivative (PDB code 6O5J (Hamiaux et al., 2019)), respectively, and found that our docking results well matched the positions of the ligands inside the receptor that were found in the experimental crystal structures (Figure S2a,b). We obtained a similar result for the structure of the ShHTL1‐glycerol complex (PDB code 5Z7W (Xu et al., 2018), Figure S2c)). For the crystal structure of ShHTL7 in complex with Triton X‐100 (PDB code 5Z95 (Shahul Hameed et al., 2018)), we found that only the part of the ligand inside the receptor pocket was docked into a position close to where it was found in the crystal structure (Figure S2d). When we compared our docking results to the experimental structure of the ShHTL7‐glycerol complex (PDB code 5Z82 (Shahul Hameed et al., 2018)), we found that in both cases, glycerol had failed to enter the ligand‐binding pocket, albeit it was located at different sites on the protein surface (Figure S2e). Encouraged by these results, we proceeded with docking studies using SL molecules as ligands. We docked 20 different SL molecules including the synthetic SL analog GR24 (Figure S1) into nine available crystal structures of SL receptors, refined the docking results with molecular dimensions (MD) simulations, and examined if the oxygen atom that is connected with a double bond to the C‐5′ position of the D‐ring would come close enough to the active site serine to be susceptible for a nucleophilic attack, which is the widely accepted first step of the SL hydrolysis reaction (Hamiaux et al., 2012; Kagiyama et al., 2013; Nakamura et al., 2013; Zhao et al., 2013), (Figure S3)).

### 3.2 A bottleneck inside the receptor acts as a size filter for large SL molecules

**FIGURE 1:**
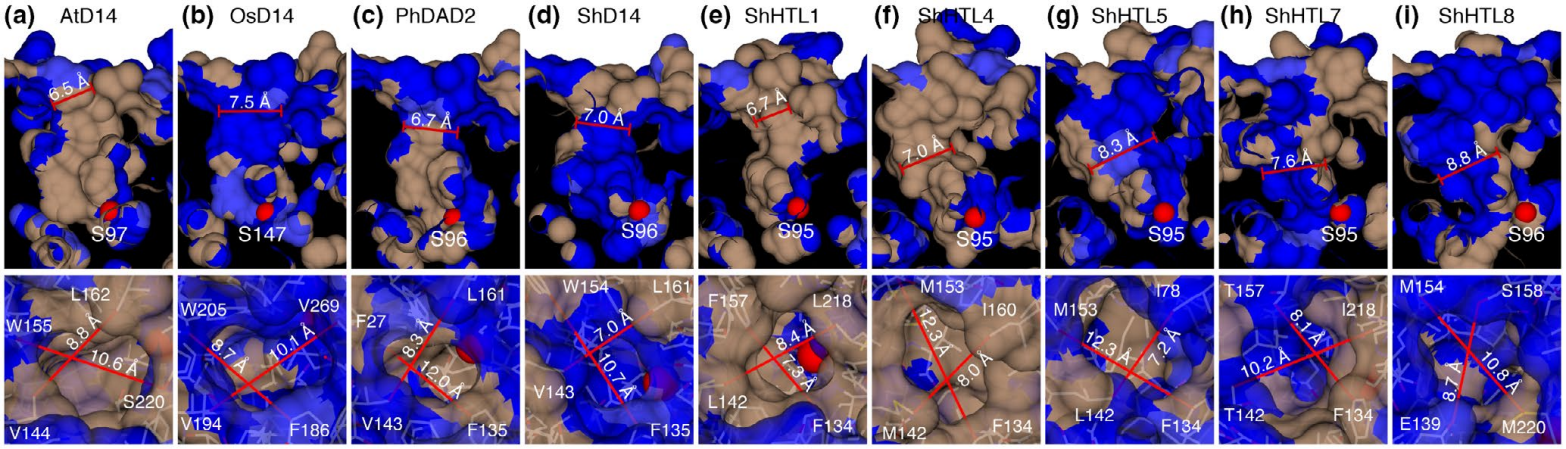
SL receptor entrances are wider than bottlenecks inside the proteins. Upper panels: Narrowest points inside the SL receptors, bottom panels: receptor entrances. (a) *A. thaliana* D14 (PDB code 4IH4); (b) *O. sativa* D14 (PDB code 3W04); (c) *P. hybrida* DAD2 (PDB code 4DNP); (d) *S. hermonthica* D14 (PDB code 5Z7Z); (e) *S. hermonthica* HTL1 (PDB code 5Z7W); (f) *S. hermonthica* HTL4 (PDB code 5Z7X); (g) *S. hermonthica* HTL5 (PDB code 5CBK); (h) *S. hermonthica* HTL7 (PDB code 5Z7Y); (i) *S. hermonthica* HTL8 (PDB code 6J2R). Protein surfaces were visualized with CCP4mg and active site serine residue oxygen atoms colored in red. Blue: hydrophilic surfaces, brown: hydrophobic surfaces

**FIGURE 2:**
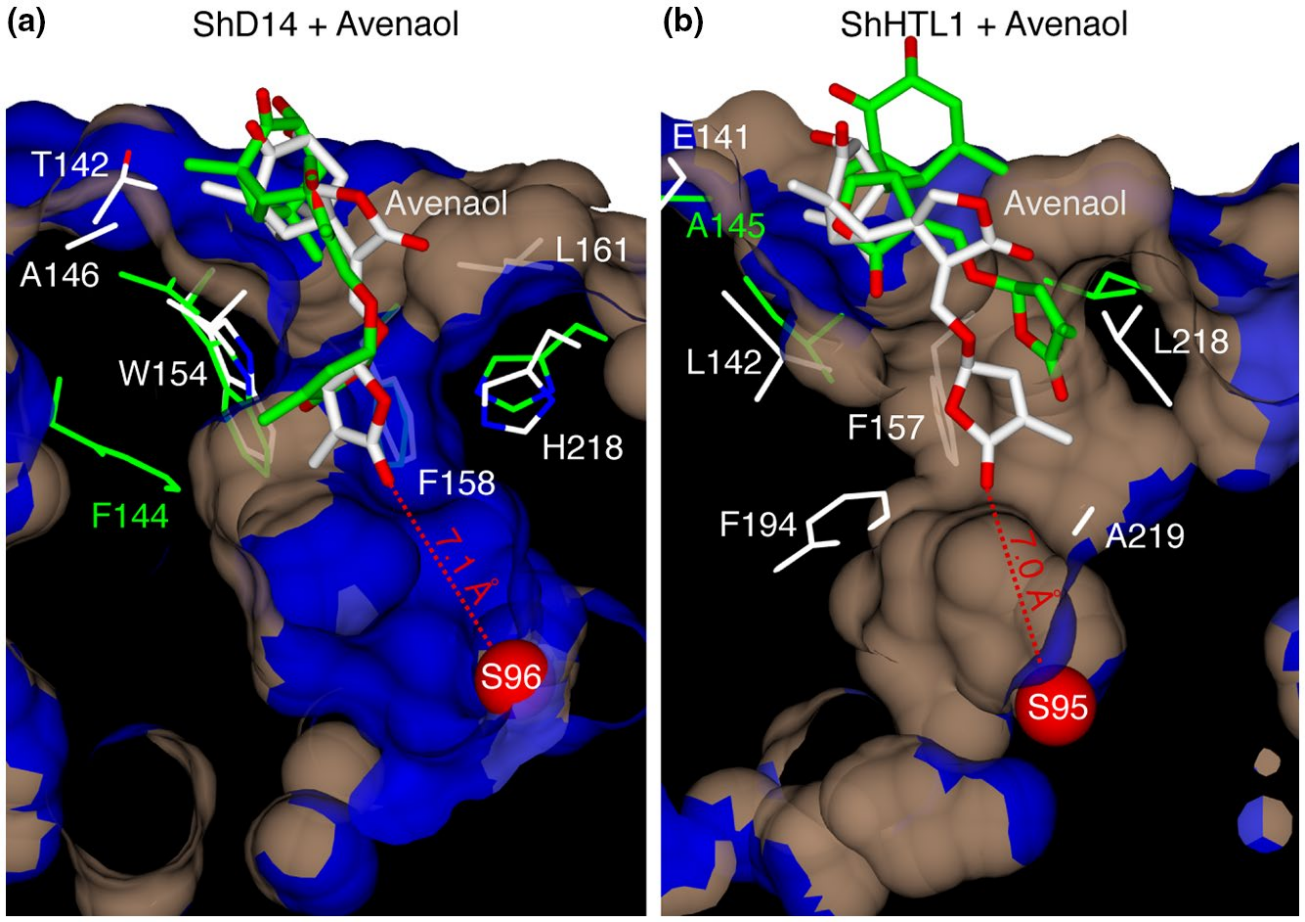
SL receptor bottlenecks serve as size filters for large SLs. Molecular docking (white) and subsequent molecular dynamics (green) of avenaol into (a) ShD14, (b) ShHTL1. Distances are shown between the oxygen atom of the SL molecule and the active site serine residue oxygen (red spheres). Blue: hydrophilic surfaces, brown: hydrophobic surfaces

We measured the diameters of the pocket entrances of different SL receptors as well as the narrowest points inside the ligand pockets. We found that while the entrances are large enough to permit entrance of all SL molecules used in this study, the narrowest diameters inside the pockets were smaller than the receptor entrances and shorter than the widths of some of the SL molecules (Figure 1, Figure S4). We found that the bulkier non‐canonical SL molecules failed to dock into receptors with a bottleneck narrower than the diameter of the SL molecule. In particular, the widest SL molecules, zealactone and avenaol, only docked into the receptors with diameters larger than 8 Å, ShHTL5 and ShHTL8 (Table 1). We examined docking results that obtained SL molecules located at the receptor entrance in MD simulations and found that these molecules failed to enter the pockets any further, indicating a lack of flexibility in the protein backbones to significantly widen the bottleneck diameter, and that these bottlenecks essentially serve as size filter for SL molecules that exceed the receptor bottleneck in diameter (Figure 2).

**TABLE 1:** Different SL receptors have distinct specificities toward SL molecules

	AtD14	OsD14	DAD2	ShD14	ShHTL1	ShHTL4	ShHTL5	ShHTL7	ShHTL8
(+)‐GR24	3.52	3.41	3.16	5.70	4.22	6.57	3.06	3.76	4.51
(−)‐GR24	6.29	4.19	5.81	3.31	2.80	3.11	4.94	5.35	3.77
Strigol	3.19	3.90	3.23	8.35	5.11	3.33	3.41	5.65	5.27
5‐deoxystrigol	3.65	3.27	7.04	3.89	5.11	2.90	3.10	3.95	4.87
Strigone	6.43	5.34	6.71	6.41	7.08	2.87	3.43	7.86	4.89
Sorgomol	3.58	6.49	6.76	8.36	5.00	5.83	3.63	3.93	5.90
Sorgolactone	3.51	3.20	3.39	6.63	4.41	5.23	3.60	3.90	4.88
(−)‐Orobanchol	3.90	3.75	6.37	5.41	4.77	3.34	3.51	3.08	3.86
(+)‐Orobanchol			3.98						
4‐deoxyorobanchol	3.62	6.39	5.71	5.66	8.05	3.23	3.52	7.90	3.33
Fabacol	3.71	3.56	4.67	8.48	5.32	8.80	2.94	7.68	4.59
Solanacol	6.20	3.93	6.20	5.14	4.82	3.54	3.43	3.08	7.95
Medicaol	6.28	3.59	6.19	3.27	4.80	6.71	3.80	3.16	3.87
Carlactone	3.16	3.90	7.18	3.82	8.33	5.70	3.92	6.10	5.23
Carlactonoic acid	2.66	7.62	7.33	3.91	8.01	3.79	3.24	7.73	3.62
Methyl carlactonoate	2.69	6.88	7.32	3.32	8.22	3.47	3.94	6.25	5.10
Avenaol	6.93	7.49	7.19	7.05	7.10	9.12	3.05	8.63	2.57
Zealactone	6.57	7.17	6.66	8.58	6.65	5.74	3.18	4.23	3.85
Zeapyranolactone	7.34	6.67	6.89	8.44	7.11	3.80	3.21	3.74	5.67
Heliolactone	3.17	3.41	3.90	7.55	8.19	2.87	3.23	2.90	3.70
Lotuslactone	9.31	7.85	6.81	3.70	7.78	3.40	3.08	3.80	4.74

Note

Shown are distances in Å between the active site serine oxygen and the oxygen atom that is connected with a double bond to the C‐5′ position of the D‐ring. Distances under 4 Å, which would be close enough for a nucleophilic attack on the SL molecule, are highlighted in green.

### 3.3 A flexible binding pocket allows for the reorientation of SL molecules

**FIGURE 3:**
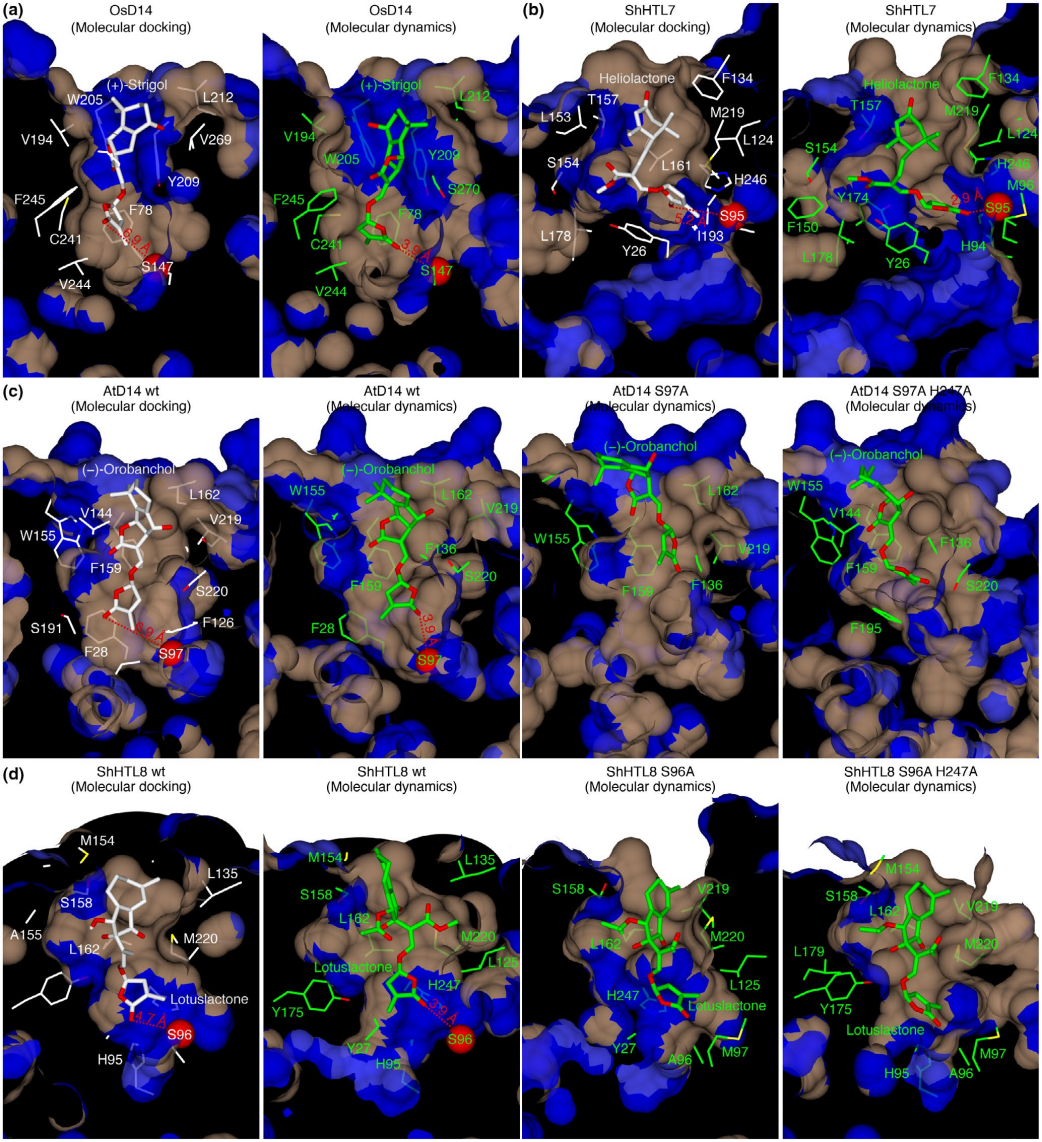
SL receptors have a flexible pocket volume that allows ligand reorientation by the active site. Molecular docking (white) and subsequent molecular dynamics (green) of (a) strigol into OsD14, (b) heliolactone into ShHTL7. (c, d) Molecular docking (white) and subsequent molecular dynamics (green) using wild‐type and active site mutant proteins of AtD14 and ShHTL8 with orobanchol and lotuslactone as ligands, respectively. Distances are shown between the oxygen atom of the SL molecule and the active site serine residue oxygen (red spheres). Blue: hydrophilic surfaces, brown: hydrophobic surfaces

We observed that in several of our docking experiments, the oxygen atom of the D‐ring was turned away from the catalytic serine, seemingly too distant for a nucleophilic attack, which is required to initiate the SL hydrolysis reaction. When subjected to MD simulations, however, in cases where the SL molecule had already fully entered the binding pocket, the ligand would rotate and stabilize with the oxygen atom turned toward the active site (Figure 3). We observed that this was accompanied by an increased volume of the binding pocket, indicating that the pockets of SL receptors are flexible enough to allow for a reorientation of the SL molecule once it has passed the initial bottleneck and successfully entered the binding site.

### 3.4 The active site plays a crucial role in ligand positioning

To examine the role of the active site in ligand binding and positioning, we repeated the MD simulations of AtD14 with orobanchol and ShHTL8 with lotuslactone using single and double mutant proteins that had the side chains of the active site serine or of the serine and histidine abolished (AtD14 S97A, AtD14 S97A H247A, and ShHTL8 S96A, ShHTL8 S96A H247A). All mutants failed to orientate the ligand the way the wild‐type proteins were able to. In the MD simulation, orobanchol in AtD14 S97A, and in AtD14 S97A H247A did rotate inside the binding pocket but stabilized with the D‐ring at a position around the center of the binding pocket (Figure 3c). In both ShHTL8 S96A and ShHTL8 S96A H247A, lotuslactone did not undergo any rotation (Figure 3d). These results suggest an important role of the active site for ligand binding before the step of hydrolysis. In particular, we observed only in the wild‐type proteins that a ligand rotation came along with a certain degree of a conformational change in the protein leading to an increased pocket volume. Our results support the observation that debranones, which are much less hydrolyzable than SLs, cause an even longer‐lasting temperature shift in DSF experiments (Seto et al., 2019). This suggests the possibility of a conformational change in the receptor upon ligand binding, which could be distinct from the conformational change observed when the receptor is bound to the F‐box protein D3/MAX2 (Yao et al., 2016).

### 3.5 Docking experiments identify a core set of amino acids involved in SL contacts

**FIGURE 4:**
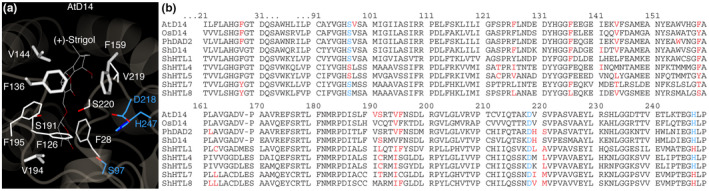
A core set of amino acids is involved in the docking of most SLs. Residues are numbered according to the *A. thaliana* amino‐acid sequence. (a) Residues making contacts with SL molecules docked in this study (strigol is shown as an example). Bold residues were found to make contacts to all docked ligands in at least seven out of nine receptors, other residues shown made contacts to all docked ligands in at least five out of nine receptors. Active site residues are colored in blue. (b) Red residues made contacts to all docked ligands in the receptor. Blue: active site residues.

To identify conserved amino acids that are a prerequisite for SL binding inside the receptor proteins, we investigated which residues in which receptors are involved in contacts to all docked SL molecules used in this study. We found a set of four amino acids that were involved in making contacts to the majority of all docked SLs into the receptors used in this study. In particular, residue F159 in *A. thaliana* and the corresponding amino acids in the other receptors contributed to the docking of all SL molecules herein. We further identified residues F136, V144, and V194 making contacts to at least 80% of all successfully docked SL molecules, and residues F28 and F126 contributing to the interaction of about half of all successfully docked SLs (Figure 4). Residues F136 and F159 are part of opposite sides of the binding pocket walls and both follow directly after glycine residues in the amino acid sequences, suggesting a certain degree of steric flexibility. Indeed, in our MD simulations, not only the position of the rotamers of these residues had changed but also the protein backbone was slightly shifted, creating a larger pocket volume.

### 3.6 Docking results suggest a variation of ligand specificity among SL receptors

Previous research has shown that SL precursors can act as signaling molecules themselves (Alder et al., 2012; Yoneyama et al., 2018) but there is no information about possible receptors of SL precursors. In addition, several plant species exude SL precursors into the soil (Wang & Bouwmeester, 2018; Yoneyama et al., 2018). The SL precursors carlactone, carlactonoic acid, and methyl carlactonoate that were included in our docking experiments are significantly more hydrophobic than the other SL molecules used herein (Figure S4). We were able to dock these three SL precursors into the *A. thaliana* SL receptor AtD14 at a distance of the D‐ring oxygen to the active site serine of less than 4 Å, and we observed the same for carlactone when docked into the rice ortholog OsD14 (Table 1). Based on our results, carlactone might also directly act as a germination signal for *S. hermonthica*, as we were able to successfully dock the molecule into ShHTL5. Carlactonoic acid could be docked into ShHTL4, ShHTL5, and ShHTL8 (Table 1). Methyl carlactonoate, the presumptive precursor of many non‐canonical SLs (Yoneyama et al., 2018), was successfully docked into ShD14, ShHTL4, and ShHTL5, susceptible to a potential nucleophilic attack (Table 1). These results suggest that several proteins might act as receptors for SL precursors, including D14 from *A. thaliana*, a species that exudes SL precursors (Wang & Bouwmeester, 2018), and receptors from *S. hermonthica*. We were not able to successfully dock as many different SL molecules into the *Petunia* SL receptor DAD2, compared to AtD14 and OsD14 (Table 1). In the SL receptor structures from the parasitic plant *Striga hermonthica*, the strongest preference for orobanchol‐type SLs was observed in ShHTL8, for which we failed to dock any strigol‐type SL. ShHTL7 seemed to have a slight preference for orobanchol‐type SLs too, although we were able to place the strigol‐type SLs sorgolactone and with some success 5‐deoxystrigol and solanacol into the binding pocket of ShHTL7. In contrast to the observed preferences in ShHTL7, ShHTL5 was able to accommodate all SLs tested in this study, except for (−)‐GR24 (Table 1). The structural reason for this lack of ligand specificity might be a combination of the large binding pocket volume, its wide bottleneck, and the availability of hydrophobic and hydrophilic patches almost throughout the entire pocket. In comparison to ShHTL5, ShHTL4 has a much more hydrophobic pocket (Figure 1f,g) and we failed to successfully dock several SLs, such as sorgomol or fabacol. We were unable to dock most SLs into ShHTL1, suggesting that the ligand for ShHTL1 might be unrelated to an SL, an unknown SL‐like molecule, or a different small molecule such as karrikins or the thus far unidentified KAI2 ligand (KL).

## 4 DISCUSSION

In this computer‐based study, we attempted to get insights into the events that take place inside the SL receptor before ligand hydrolysis. Since we were unable to directly show hydrolysis events, we judged substrate specificity based on results from molecular docking and MD simulations after which an SL molecule was positioned in a distance of its D‐ring oxygen to the active site nucleophile that would be close enough for a nucleophilic attack. The only available crystal structure of an SL receptor in complex with the intact SL analog GR24 (Zhao et al., 2015) shows an orientation of the D‐ring toward the active site. However, there is uncertainty surrounding the accuracy of the structure and whether the atomic coordinates of the ligand are supported by electron density (Bürger & Chory, 2020; Carlsson et al., 2018). Based on these docking results, we performed MD simulations to assess the mobility of the ligand and the flexibility of the receptor proteins. MD has been used before to describe intermediate stages of karrikin binding to its receptor (Hu et al., 2019), a close homolog of SL receptors, and to investigate the flexibility of specific regions in the SL receptor DAD2 that might serve as interaction interfaces to downstream partners (Lee et al., 2020).

When we assessed the initial entrance of SL molecules into different receptor proteins, we found that the entrance in the protein lid would likely not constitute a significant barrier even for the larger non‐canonical SLs, since we found all of the entrances with a width of at least 8.5 Å (Figure 1), which is wider than the most bulky SL molecule used in this study, zealactone, for which we measured a width of 8.2 Å (Figure S4). However, the narrowest points in SL receptors appear to be the bottlenecks further inside the proteins, which might restrict the movement of SLs that exceed this diameter toward the active site. In our docking studies, SL molecules larger than these bottlenecks failed to dock into the binding site. In subsequent MD simulations, these ligands did not move further toward the active site, which suggests that the receptor bottlenecks defining the narrowest parts of the SL receptors are of rather rigid nature and that they might constitute a size filter for many of the bulky non‐canonical SLs. Two studies using *Striga* (Xu et al., 2018) and *Physcomitrella* (Bürger et al., 2019) proteins have demonstrated that the binding pocket diameter is one important feature for ligand specificity and that it is, indeed, a rather rigid feature under control of hydrogen bonds located outside of the pocket. This could be especially important for some of the larger non‐canonical SLs, some of which are strong inducers of *Striga* germination (Yoneyama et al., 2018) One might speculate that some large non‐canonical SL molecules, if taken up by other plants, may potentially act as allelochemicals, binding to narrow SL receptors without reaching the active site, thus failing to trigger the signaling state. Their potential competition with the intrinsic SLs for the same receptor could, therefore, interfere with SL signaling and lead to impaired shoot growth in the receiver plant.

Our molecular dynamics simulations suggest that in case a ligand has successfully entered the binding site, it might undergo reorientation and stabilize in a position with the oxygen atom of the D‐ring oriented toward the active site serine, which would be a prerequisite for a nucleophilic attack and subsequent SL hydrolysis. We observed that these reorientations coincided with an increased volume of the binding pocket, suggesting that the binding pocket has sufficient intrinsic flexibility for ligand reorientation and that the pocket volume itself might not be a restricting factor for ligand specificity, because it can be adjusted. In a recent MD study that investigated SL binding to AtD14 and ShHTL7, a flexible ligand‐binding pocket was observed, too, which seemed to be regulated through a hinging motion between helices T1 and T2 in the protein lid (Chen, White, Nelson, & Shukla, 2020). One crystal structure is available that provides a catalytic mutant of an SL receptor, DAD2 S96A. The structural difference to the wild‐type protein is minor, resulting from the loss of a hydrogen bond between the oxygen in S96 and the main chain nitrogen in F27 (Hamiaux et al., 2012). Since these structures do not contain a substrate, we attempted to probe the importance of the active site for the correct positioning of the SL molecule. We found that both side chains of the catalytic serine and histidine were essential for ligand positioning. This underscores a potential dual role of the active site in SL receptors, ligand binding, and ligand hydrolysis. This dual role is not necessarily typical. For example, the enzyme acetylcholinesterase is also an α/β hydrolase with a hydrophobic binding pocket and an intact catalytic triad. However, the active site does not seem to significantly contribute to substrate binding and in fact, crystal structures are available of catalytically dead receptor‐ligand complexes (Bourne et al., 2006). This highlights the challenge of performing studies using catalytic mutants of SL receptors in general because it can be difficult to discriminate between ligand binding and ligand hydrolysis. It has been shown using differential scanning fluorimetry (DSF) that receptor destabilization took place when a non‐hydrolyzable SL analog was used and that it did not take place when catalytic serine to alanine mutants were used (de Saint Germain et al., 2016). Our results might support the idea of a pre‐hydrolysis receptor destabilization and conformational change upon ligand binding, which is caused by an increased pocket volume and ligand movement. We attempted to identify a core set of conserved amino acids that may be essential for the binding of different SL molecules. We found that residues F136 and F159 (*A. thaliana* nomenclature) are part of the binding pocket wall and were involved in making contacts to SL molecules in all of our docking experiments where an SL had successfully entered the binding pocket. The fact that these residues are immediately adjacent to glycines might suggest intrinsic flexibility of the binding pocket, since glycine residues display extraordinary flexibility due to their lacking side chain (Richardson & Richardson, 1988).

In this study, we also attempted to assess the ligand specificity of nine different SL receptors from the model organisms *Arabidopsis*, rice and *P* *etunia* as well as from the parasitic plant *S. hermonthica*. We observed that in our docking and MD simulations, both strigol‐type and orobanchol‐type SLs could be successfully placed into AtD14 and OsD14, albeit the fact that rice only produces orobanchol‐type SLs (Xie et al., 2013; Yoneyama et al., 2018), at least judged by those molecules that have been identified from root exudates so far. We were unable to dock any orobanchol‐type SLs into *Petunia* DAD2. It has been previously demonstrated that DAD2 is able to bind a racemic mixture of orobanchol when tested in differential scanning fluorimetry (DSF) assays (Hamiaux et al., 2018), in our docking experiments, however, we were only able to dock the non‐natural (+)‐orobanchol into DAD2 (Table 1).

We observed significant variation in ligand binding by different SL receptors from *S. hermonthica*. While ShHTL5 turned out to be the most promiscuous receptor, ShHTL8 would not accept any strigol‐type SLs in our docking studies. In a previous report, ShHTL7 was shown to be the most sensitive SL receptor in *S. hermonthica* when examined in germination assays using different SL molecules (Toh et al., 2015). We were able to dock some but not all of the SLs used in this study into ShHTL7; however, the germination assays were performed with racemic mixtures and our docking experiments were run with all SL molecules in the 2′*R* configuration of the enol‐ether bridge. In addition, we did not succeed to dock any SL except (−)‐GR24 into ShHTL1, which is in agreement with the results from the germination experiments in the previously mentioned study (Toh et al., 2015). The observed variation seems to hold true for a certain selectivity toward non‐canonical SL molecules, too. The SL precursors carlactonoic acid and methyl carlactonoate have been reported in the root exudate of *Arabidopsis* (Wang & Bouwmeester, 2018) and we were able to successfully dock these two molecules and carlactone into AtD14. The most promiscuous SL receptor, ShHTL5, was able to accommodate all non‐canonical SLs tested in this study, whereas other *S. hermonthica* receptors appeared more specific, such as ShHTL8 for avenaol and zealactone and ShHTL4 for zeapyranolactone and lotuslactone.

Our study suggests that *S. hermonthica* might have developed both promiscuous SL receptors that perceive almost all SL molecules tested herein, and specific ones that display a narrower recognition toward SLs produced by certain hosts or SLs from a certain type. This might have led to increased fitness of *S. hermonthica* because it can modulate specific responses toward individual hosts and at the same time express receptors for SL molecules for which no specific receptor has evolved yet. Hopefully, biochemical experiments will be able to relate our results to affinities between SL molecules and different receptors. It remains unclear whether the germination of *Striga* seeds can be stimulated by SL binding to a single receptor or if integration of several receptor signals is required. It will also be interesting to see how receptor specificities differ from those in *Striga asiatica*, a parasitic plant that has 17 potential SL receptors (Yoshida et al., 2019), or from other root parasitic plants, such as*Phelipanche*, which has recently been demonstrated to possess at least one receptor with enzymatic activity toward SLs (de Saint Germain et al., 2020).

## CONFLICT OF INTEREST

The authors declare no conflict of interest associated with the work described in this article.

## AUTHOR CONTRIBUTIONS

M.B. and J.C. designed the research, M.B. performed the research, and M.B and J.C. wrote the manuscript.

## Supporting information

Supinfo S1
