# Supplementary material for: In‐silico analysis of the strigolactone ligand‐receptor system
Source: Plant Direct. 2020 Sep 15;4(9):e00263. doi: 10.1002/pld3.263 (PMC7507525; doi:10.1002/pld3.263)
Supplement: Supplementary file 1 — Supinfo S1 [file PLD3-4-e00263-s001.pdf]

Supplement

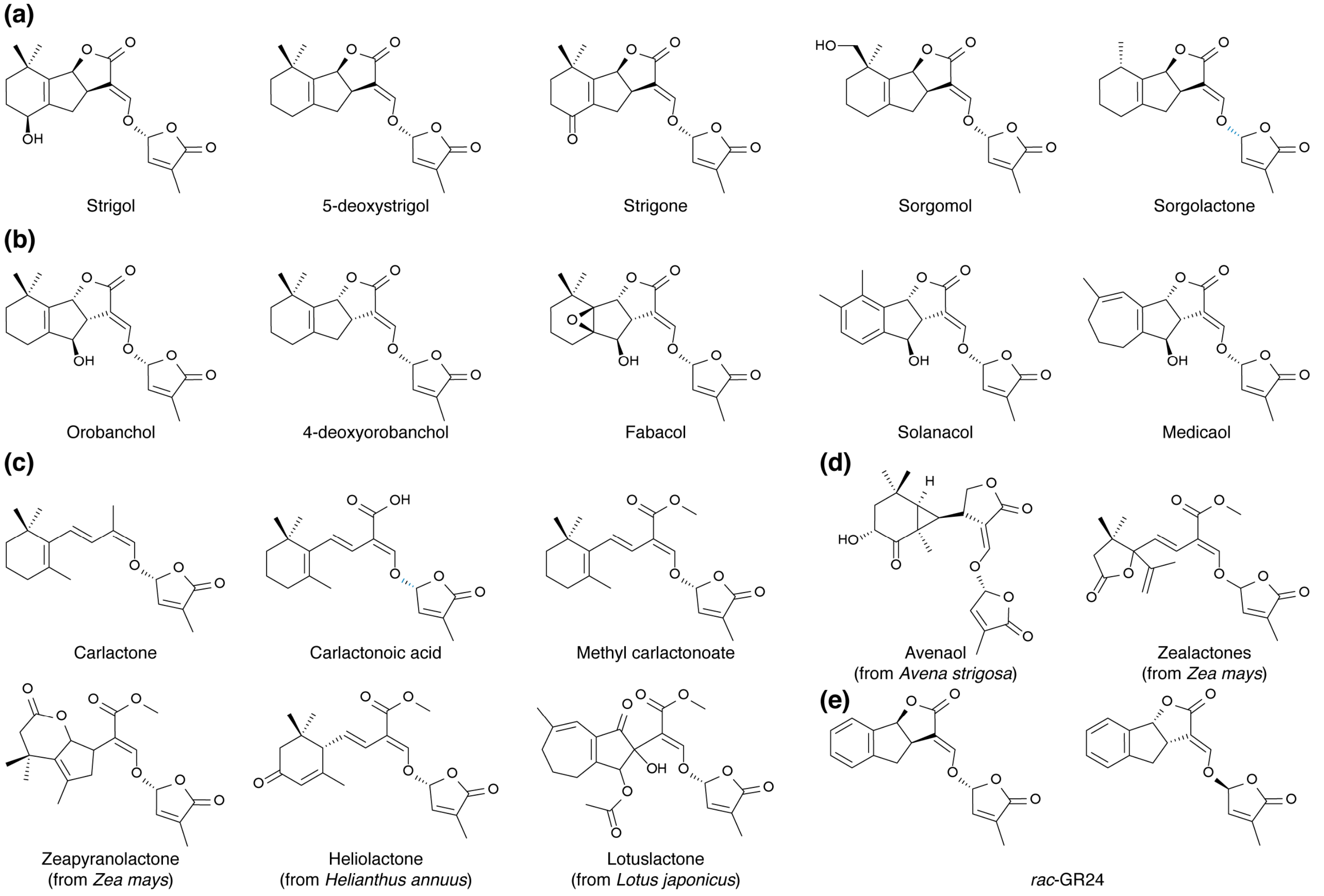

Figure S1: SL molecules used in this study. (a) strigol-type SLs, (b) orobanchol-type SLs, (c) non-canonical SLs that are SL precursors, (d) other non-canonical SLs, (e) the synthetic SL analog GR24.

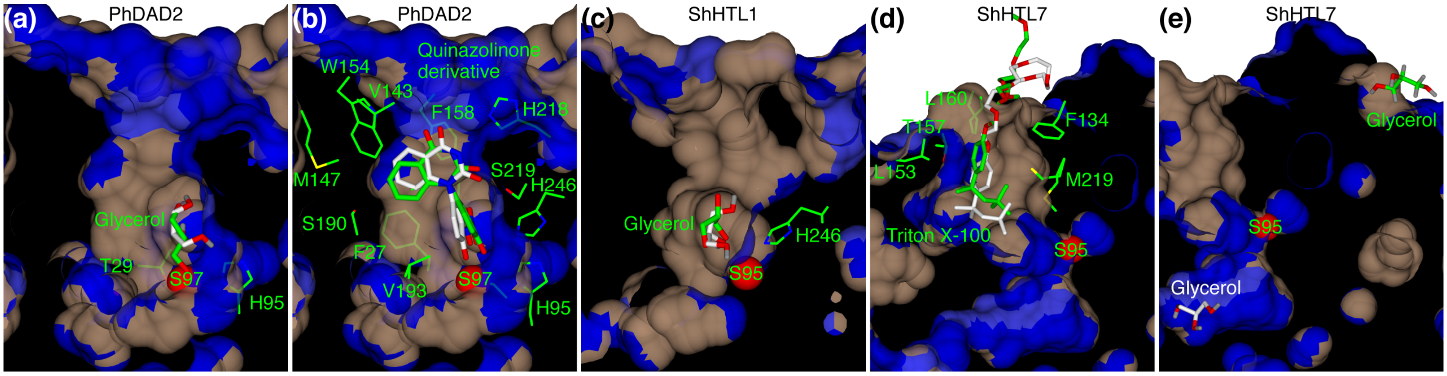

Figure S2: Experimental crystal structures (white) and molecular docking (green) of (a) PhDAD2 in complex with glycerol (PDB code 4DNP), docking affinity was -3.5 kcal/mol. (b) PhDAD2 in complex with a quinazolinone derivative (PDB code 6O5J), docking affinity was -5.9 kcal/mol. (c) ShHTL1 in complex with glycerol (PDB code 5Z7W), docking affinity was -3.5 kcal/mol. (d) ShHTL7 in complex with Triton X-100 (PDB code 5Z95), docking affinity was -5.2 kcal/mol. (e) ShHTL7 in complex with glycerol (PDB code 5Z82), docking affinity was -3.5 kcal/mol. Protein surfaces were visualized with CCP4mg. Blue: hydrophilic surfaces, brown: hydrophobic surfaces.

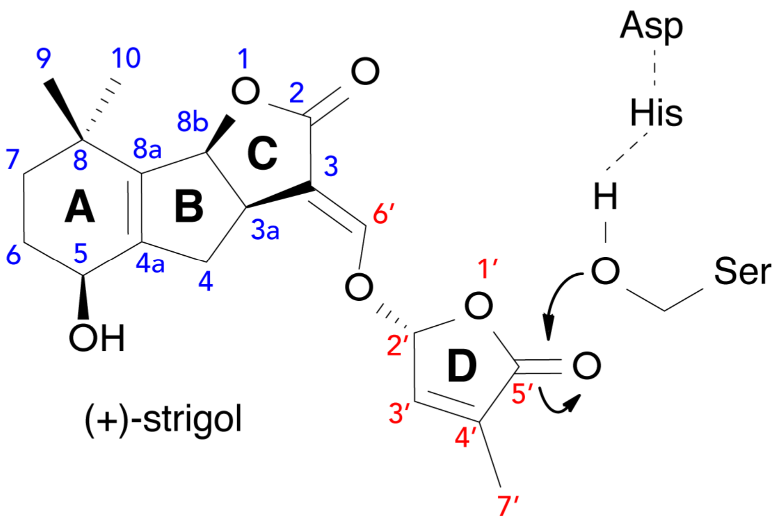

Figure S3: First step of D14/DAD2/HTL-mediated SL hydrolysis. The reaction begins with the well-accepted nucleophilic attack by the serine residue in the catalytic triad.

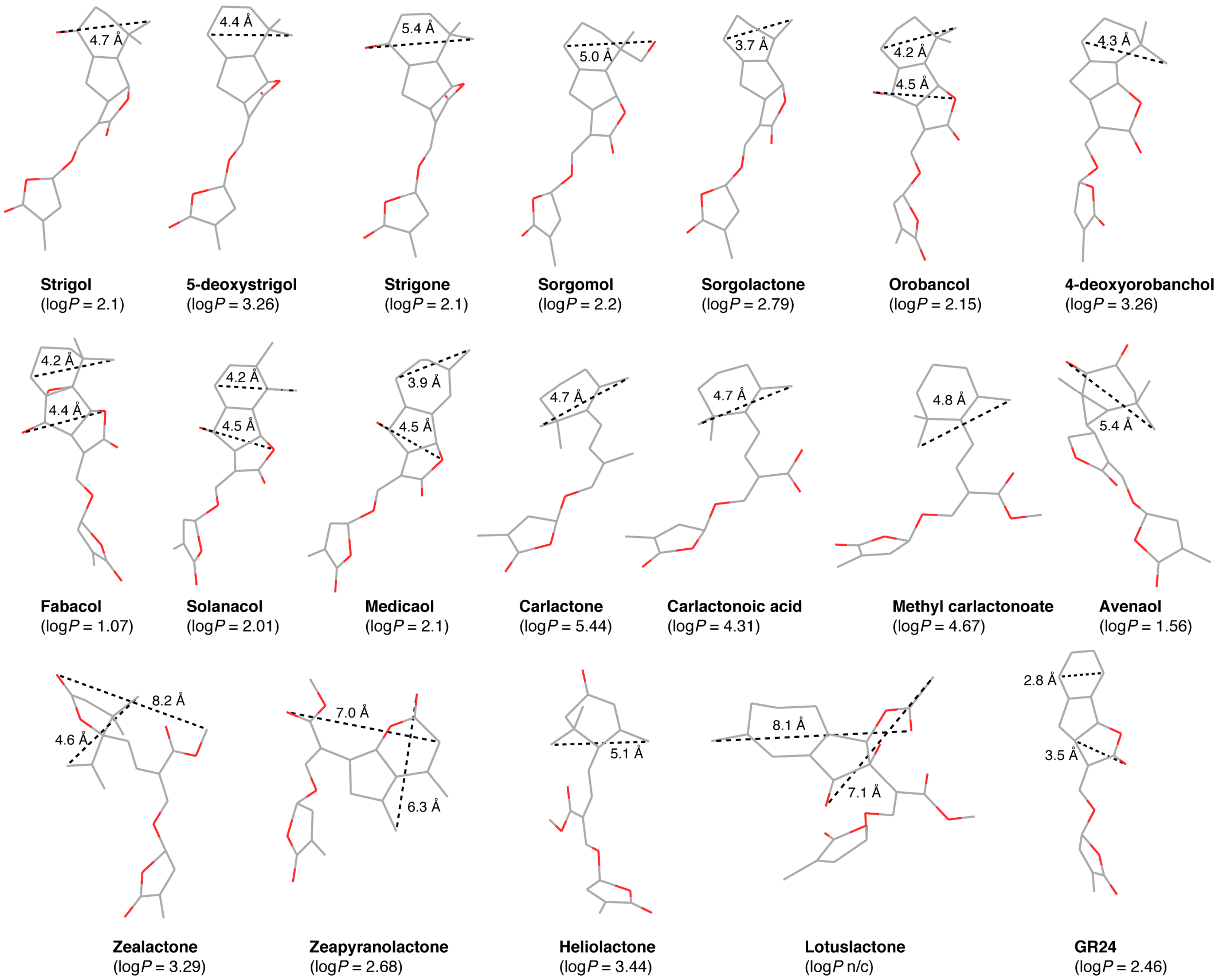

Figure S4: Diameters and hydrophobicity of SL molecules used in this study. Log*P* values were calculated with ChemDraw (PerkinElmer), and higher values indicate a more hydrophobic compound (logarithmic scale). Distances between atoms were measured with Chem3D (PerkinElmer).

Table S1: Affinities from the docking experiments. Values are in kcal/mol.

	AtD14	OsD14	DAD2	ShD14	ShHTL1	ShHTL4	ShHTL5	ShHTL7	ShHTL8
(+)-GR24	-9.2	-8.1	-6.2	-9.2	-7.2	-7.4	-8.4	-7.8	-8.1
(–)-GR24	-9.4	-9.5	-6.3	-8.6	-7.3	-8.9	-8.2	-7.4	-8.3
Strigol	-7.9	-8.7	-6.2	-7.8	-6.7	-7.5	-9.3	-6.9	-7.9
5-deoxystrigol	-7.6	-8.8	-7.7	-7.6	-7.5	-7.4	-9.5	-8.4	-7.3
Strigone	-9.8	-8.7	-6.9	-7.6	-8.2	-7.4	-9.9	-7.4	-8.7
Sorgomol	-8.3	-9.0	-6.3	-7.9	-7.4	-6.5	-9.1	-7.8	-7.5
Sorgolactone	-9.6	-9.9	-6.9	-8.2	-7.5	-7.0	-9.1	-8.1	-8.9
(–)-Orobanchol	-7.9	-8.4	-7.1	-7.6	-7.3	-6.4	-8.9	-8.1	-6.5
(+)-Orobanchol			-7.5						
4-deoxyorobanchol	-8.3	-8.3	-7.9	-9.1	-7.2	-8.0	-9.1	-7.5	-8.0
Fabacol	-7.8	-9.0	-7.3	-7.5	-7.5	-7.6	-9.8	-7.9	-8.0
Solanacol	-8.2	-8.3	-7.6	-10.0	-6.9	-7.2	-8.8	-8.3	-6.5
Medicaol	-8.6	-8.8	-7.3	-8.2	-7.1	-7.1	-8.7	-8.2	-6.7
Carlactone	-8.6	-8.1	-7.3	-7.9	-6.3	-8.6	-8.8	-7.0	-7.9
Carlactonoic acid	-8.3	-7.5	-7.4	-7.2	-5.8	-7.1	-7.9	-7.4	-7.0
Methyl carlactonoate	-8.2	-6.7	-7.1	-6.5	-6.1	-7.2	-8.1	-7.7	-8.0
Avenaol	-8.0	-8.6	-7.9	-7.2	-7.1	-7.1	-8.9	-7.0	-8.6
Zealactone	-7.0	-7.1	-6.8	-6.9	-5.7	-7.1	-8.2	-6.7	-7.6
Zeapyranolactone	-7.8	-7.8	-6.9	-7.1	-7.5	-6.2	-8.8	-7.6	-7.1
Heliolactone	-8.1	-7.5	-5.4	-7.2	-5.6	-7.3	-7.5	-6.4	-7.6
Lotuslactone	-6.3	-7.2	-6.3	-5.6	-5.3	-5.7	-8.7	-7.5	-6.8
